# Hemorrhage following Abortion Readily Controlled by the Indicated Remedy

**Published:** 1893-04

**Authors:** J. A. Tomhagen

**Affiliations:** Philadelohia, Pa.


### HEMORRHAGE FOLLOWING ABORTION READILY CONTROLLED BY THE INDICATED REMEDY.

J. A. Tomhagen, M. D., Philadelphia, Pa.

Case I.—Mrs. E. H., æt. twenty-four. Menses absent two months and positive she was pregnant. Has taken many drugs and teas to rid herself of the fœtus. March 4th, 1889, she rode a horse about three miles, and noticed that she was flooding before she reached her destination. She was put to bed, and after several severe pains the fœtus was extruded. The hemorrhage was profuse and painless and bright red. When I arrived her face was blanched, and she frequently gasped for breath. The slightest motion would cause a gush of blood, which was followed by a deathly sinking at the epigastrium.

Ipec.$^{200}$ controlled the hemorrhage at once. The woman experienced a peculiar, indescribable sensation pass over her in a few seconds. The gushes then came at longer intervals and the fainting ceased immediately. The one dose was all-sufficient.

Case II.—Mrs. F. G., æt. twenty-eight. Blonde. This woman maintained that the "miscarriage" came on spontaneously; she could account for it in no way except that carrying a bucket of water caused it. She had been flooding *in spells* for several days, when I saw her, May 13th, 1889. Blood dark and clotted. As soon as she saw me, she began *to weep*, but could say nothing; moreover, she was as patient as a lamb; her mouth was *parched*, and yet she desired *no water*. The fœtus of a few weeks must have been buried with the clots since I could find none. Puls.$^{2m}$.

In five minutes a sensation as if the *whole external genitals were drawn up into the pelvis* followed. (Puls.$^{10m}$ has produced this several times. In fact, one girl remarked that I had given her dynamite, the action was so violent.) With this sensation the hemorrhage ceased and returned only slightly in six hours. No more medicine.

Case III.—Mrs. J. S., æt. twenty. Married a short time; got into an altercation with another of the fair sex. Both being typical brunettes, they well-nigh demolished each other. My patient tore and swore, fumed and stamped, when separated, because she was not permitted to annihilate her adversary; she retired very disconcerted, and during the night was taken with violent cramps and vomiting of bile; intense bearing-down pains and sharp, excruciating pain in the left ovarian region; she could not lie still a moment, and the only relief was obtained from hard pressure and hot fomentation on abdomen. The hemorrhage was slight, two A. M., Coloc.$^{3m}$.

In twenty minutes the pains abated and hemorrhage stopped.

Six A. M., very comfortable, but face as yellow as a lemon; no more pain and carried child to full term.

*Remarks.*—The single dose of the appropriate remedy was all-sufficient in these cases.

Some maintain that it is criminal to rely on medicine in abortion. It is criminal to do other than give the indicated remedy.

The above cases I regard as typical of abortion.

Am., Gels., Sabina, Apis, etc., do the work as effectually, when called for.

China often removes membranes that have kept up flooding for weeks, and then the hemorrhage ceases.

Pyrogen. cured several cases where symptoms of septicæmia supervened. The China and Pyrogen. cases come from the scientific doctors.

Dynamics supersede mechanics.

